# Pirenzepine Binding Sites in the Brain of the Honeybee *Apis mellifera*: Localization and Involvement in Non-Associative Learning

**DOI:** 10.3390/insects13090806

**Published:** 2022-09-05

**Authors:** Chaïma Messikh, Monique Gauthier, Catherine Armengaud

**Affiliations:** 1 Centre de Recherches sur le Cognition Animale (CRCA), Centre de Biologie Intégrative (CBI), Université de Toulouse, CNRS, UMR 5174-CNRS, -IRD, UPS, 31062 Toulouse, France; 2 Laboratoire Evolution et Diversité Biologique (EDB), Université de Toulouse, UMR 5174-CNRS, -IRD, UPS, 31062 Toulouse, France

**Keywords:** honeybee, brain, pirenzepine, muscarinic receptors, PER, habituation

## Abstract

**Simple Summary:**

The cholinergic system has been extensively studied in insects as the target of pesticides that affect the functioning of the nicotinic acetylcholine receptors. In the honeybee, the detrimental effects of pesticides are observed in behavior, including associative learning. The current work describes the expression of muscarinic acetylcholine receptors in the honeybee brain. We show that the muscarinic antagonist pirenzepine induces a slow-down of non-associative learning, the habituation of the proboscis extension reflex. We suggest that pirenzepine binding sites located on the neurons of the subesophagous ganglion are involved in the habituation of the reflex.

**Abstract:**

Muscarinic acetylcholine receptors (mAChRs) play a central role in learning and memory in mammals as in honeybees. The results obtained in the honeybee *Apis mellifera* are based on the detrimental effects of the mAChR antagonists, atropine and scopolamine, on olfactory associative memory. Binding sites for the mAChR antagonist BODIPY^®^ FL pirenzepine were localized in the brain of the honeybee forager. Pirenzepine binding sites were detected indifferently in several somata and neuropilar areas. The highest binding site densities were present in the central complex and in somata of the dorsomedial border of the antennal lobes. An additional binding pattern was found in somata of the subesophageal ganglion. By contrast, Kenyon cell (KC) somata were not stained. Pirenzepine (PZ) effects on non-associative learning were evaluated. Treated animals required more trials for the habituation of the proboscis extension reflex (PER) than controls, and the duration of the PER increased after PZ brain injection. These results suggest that the network mediating habituation of the PER involves PZ binding sites that are not necessarily present on the circuitry mediating olfactory conditioning of the PER.

## 1. Introduction

The cholinergic system has been extensively studied in insects as the target of many pesticides. The pesticides affect the cholinergic neurotransmission essentially by altering the functioning of the nicotinic acetylcholine receptors [1]. Although muscarinic receptors could open new prospects to insect repellents [2], this class of receptors is the subject of few studies of insects. In the honeybee *Apis mellifera*, both muscarinic and nicotinic binding sites have been described in the brain. Data have shown that muscarinic receptors are 3.6 times less abundant than nicotinic receptors [3], and a detailed mapping of muscarinic binding sites in the brain of this insect is still missing. Some studies on muscarinic acetylcholine receptors (mAChRs) have been performed in various insects. These receptors were divided into three subfamilies named A, B and C, that have been previously characterized in *Drosophila melanogaster* [4,5,6]. The transcripts of all three mAChR genes are highly expressed in the brain of the agricultural pest *B. dorsalis* [7]. The A- and C-types are present in the honeybee [4,7] and the A-type is found only in the brain of the oriental armyworm *M. Separata* [8]. The A- and C-types, such as the mammalian M1/M3/M5 receptors, which signals via Gq/11, are generally excitatory, and the B-type receptor, such as M2/M4 receptors, which signals via Gi/o, is generally inhibitory [4,6]. The classical muscarinic antagonists, atropine, scopolamine, and 3-quinuclidinyl-benzilate (QNB), block the A-type, while these antagonists do not block the B-type [4].

The effects of pirenzepine (PZ) and those of other muscarinic antagonists have been examined on the neuronal electrical responses in several insect species (for a review, see [9]). In the moth *Manduca sexta*, PZ abolishes the excitatory response of a motoneuron to sensory afferent stimulation [10]. In the locust and cockroach, PZ seems to be a very selective inhibitor of the depolarizing component of the induced muscarinic response [11,12]. In the cockroach dorsal unpaired median (DUM) neurons, the nicotinic [Ca^2+^]i increase is reduced by the nicotinic antagonist α-bungarotoxin, and also by PZ [13], indicating that ‘mixed nicotinic–muscarinic receptors’ exist and could be the target of PZ. It was also reported that PZ could inhibit the modulatory effects of muscarinic receptors on nornicotine-induced currents [14]. 

Behavioral studies in *Drosophila* have shown that the aversive olfactory learning requires A-type mAChRs, located in the gamma subtype of Kenyon cells (KC) of mushroom bodies (MB) [15]. Evidence for muscarinic-like receptors’ involvement in appetitive olfactory memory has been presented in the honeybee. The muscarinic antagonist scopolamine injected into the brain, or into the MB, inhibited the retrieval of the olfactory conditioning of the proboscis extension reflex (PER), whereas scopolamine had no effect on the acquisition and storage processes of the olfactory conditioning [16,17]. The same observations were reproduced with atropine, but PZ was without effect even at a dose of 10^−2^ M [18]. 

We then raise the questions of whether PZ binding sites are expressed in the mushroom bodies of the adult bee and if they play a role in learning. Several muscarinic receptor subtypes have been proposed in the honeybee [4], including one showing a low-affinity binding site for the vertebrate M1 antagonist, PZ [19]. Bodipy PZ derivatives were found to be valuable tools to bind to both the orthosteric and allosteric receptor sites of the human M1 mAChR [20]. In the present paper, a fluorescent Bodipy PZ was used to visualize muscarinic-like receptors in the honeybee brain. Our results are the first to report the presence of PZ binding sites in somata and neuropilar regions involved in sensory, motor and cognitive processes. We then address the question of the involvement of PZ in non-associative learning in the honeybee. To do that, PZ was injected into the brain of the insect and the effects were assessed for the habituation of the PER.

## 2. Materials and Methods

### 2.1. Distribution of BODIPY^®^ FL Pirenzepine Binding Sites

Worker bees (*Apis mellifera*) were caught at the hive entrance. The head was cut, and the brain was rapidly dissected. The brain was fixed in 4% phosphate-buffered paraformaldehyde (pH 7.4) for 1 h, rinsed in PBS and cryoprotected overnight in a solution of sucrose (20%). Serial cryostat sections (16 μm) were made in the three major planes (frontal, sagittal and horizontal), and then mounted on gelatin-coated slices. 

Sections were incubated with 10^−8^ M or 10^−6^ M BODIPY^®^ FL pirenzepine (Molecular Probes, Eugene, OR, USA) and dissolved in PBS for 1 h at room temperature in darkness. After incubation, the sections were washed twice for 10 min in PBS, rapidly dipped in cold water and mounted in Mowiol. BODIPY^®^ green fluorescence was observed with a Zeiss Axoplan microscope. Sections were initially viewed using a FITC filter (Zeiss, Jena, Germany) and 20×, 40× water-immersion objectives and a 100× oil-immersion objective, then photographed (Kodak ASA 400). Digital images were captured and processed to black and white pictures in Adobe Photoshop 7 to provide identification of brain structures and somata on the pictures.

Competitive displacement experiments were conducted on sections that were preincubated for 1 h with 10^−2^ M or 10^−3^ M unlabeled pirenzepine (Sigma-Aldrich, St Quentin Fallavier, France) and then incubated with 10^−6^ M BODIPY^®^ FL pirenzepine in the presence of 10^−2^ M pirenzepine. A competitive experiment with unlabeled scopolamine (Sigma-Aldrich) 10^−2^ M applied over a 2-h period was less effective in displacement than PZ (Figure S1). In total, 75 brains were prepared for incubation with 10^−6^ M BODIPY^®^ FL pirenzepine. Brains were dedicated to the development of the technique and the experiments of displacement with unlabeled ligands. Only half were used for localization of somata.

### 2.2. Behavioral Tests

Bees were collected in the morning (9–9.30 AM) and transported to the laboratory in glass tubes. The tubes were placed in ice for 5 min to immobilize the bees. Then, they were placed in small tubes and fixed with a tape and a drop of wax on the thorax. The animal could move only its antenna and proboscis. Before the injections began, a median ocellar dissection was done. A time interval of 30–45 min was left between the dissection and injection to allow the bees to recover from the operation. A volume of 0.4 μL PZ (10^−1^, 10^−2^ or 10^−3^ M in PBS) was injected into the ocella with the aid of a micro-syringe (Hamilton company, Reno, NV, USA). The PBS solution was injected in bees of the control group (0 μg/bee); the PZ groups were injected in parallel. 

These experiments are conducted using sucrose or fructose as antennal stimuli because appetitive olfactory learning is affected by sugar identity [21]. Both sugars are important for bee nutrition. Nectar is mainly composed of sucrose and a lower proportion of other sugars, such as fructose. Fructose is one of the main sugars in honey and an important food source for bees. Sugar solutions (sucrose or fructose, 50% *w*/*v*) were used to stimulate the antennae and to feed the bees after the tests. We also counted the number of dead honeybees 24 h after injection in the control and PZ-injected animals to eliminate a possible toxic effect of pirenzepine. 

#### 2.2.1. Habituation

As a learning procedure, we used the habituation of the PER previously studied in the honeybee [22,23]. The habituation task was performed 2 h after ocellar injection. Fasted honeybees were repeatedly stimulated in 6 s intervals with a 50% (*w*/*v*) sugar solution (sucrose or fructose) applied to one antenna for one second. The sugar solution induced the PER, and the bees were not allowed to consume sugar. The habituation criterion was defined as three consecutive antennal stimulations that did not induce the PER. A restoration test was carried out after the honeybee attained the habituation criterion to exclude the possibility of motor fatigue by applying the sugar solution to the contralateral antenna. Bees that did not respond to the 50% sugar solution at the start of the training session and to the PER restoration test at the end of the session were discarded.

#### 2.2.2. Duration of Proboscis Extension

In order to evaluate the duration of the proboscis extension, a 10^−3^ M sugar solution was used to stimulate the antennae 1 h, 2 h, and 3 h after PZ or PBS injection. During this test, only two stimulations were performed on each antenna with either sucrose or fructose (50% *w*/*v*). The duration of the PER was measured with a digital timer.

### 2.3. Data Analysis

Habituation data were analyzed with one-way ANOVA. Post hoc comparisons were carried out when allowed using Scheffe tests. To detect a potential effect of PZ on the duration of the PER a two-way ANOVA test (group effect: control, PZ; time effect: 1 h, 2 h and 3 h) and the Scheffe post hoc test were performed. Data are presented as mean ± SEM. The percentage of dead bees was also calculated 24 h after injection. In this case, χ^2^ tests were used to compare the control and treated groups. A *p* value < 0.05 was considered significant. 

## 3. Results

### 3.1. Visualization of BODIPY^®^ FL Pirenzepine Binding Sites in Honeybee Brain Slices

#### 3.1.1. Antennal Lobes

**Figure 1:**
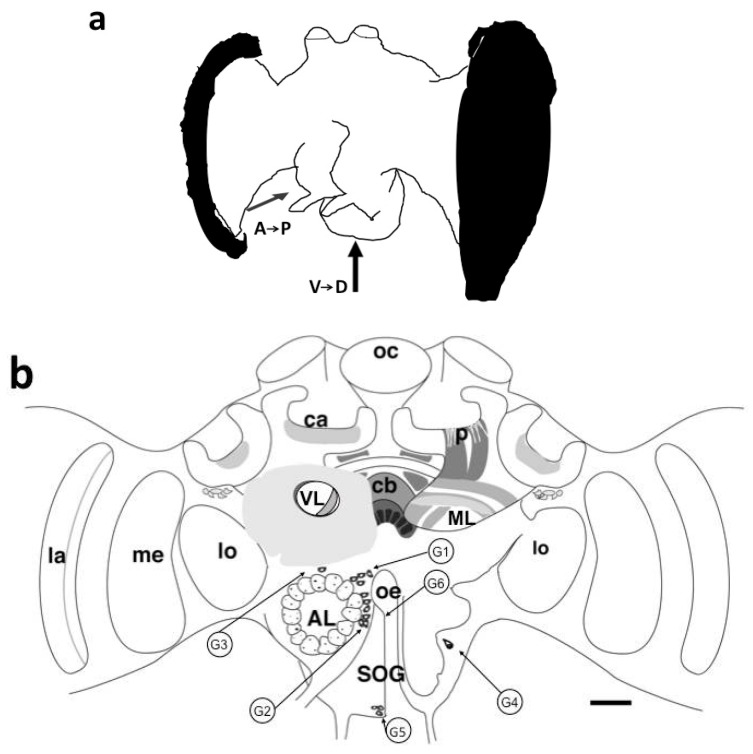

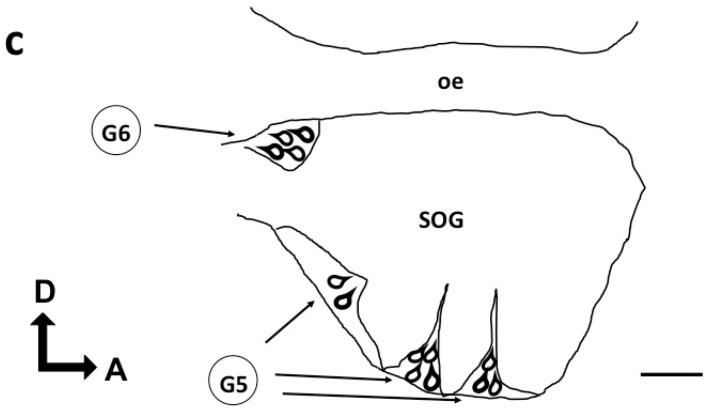
(**a**). Drawing of a 3D honeybee brain. Serial cryostat sections were made in the frontal, sagittal and horizontal planes. Stained somata (G1–G6) were identified by the depth calculated from the surface of the antennal lobe in the antero–posterior axis from frontal sections and the height calculated from the lower part of the subesophageal ganglion in the ventro–dorsal axis from horizontal sections as indicated on Table 1. (**b**): Schematic representation of BODIPY^®^ FL Pirenzepine binding sites in frontal sections of the honeybee brain. Oc: ocelli, ca: calyx, p: peduncle, cb: central body, VL: vertical lobe, ML: medial lobe, AL: antennal lobe, DL: dorsal lobe, OT: optic tubercle, lo: lobula, me: medulla, la: lamina, SOG: subesophageal ganglion. Stained somata (G1–G6). Scale = 100 μm. (**c**): Drawing of stained somata of the G5 and G6 groups in the subesophageal ganglion (SOG) sectioned in the sagittal plane. oe: esophagus. Scale = 100 μm.

**Figure 2:**
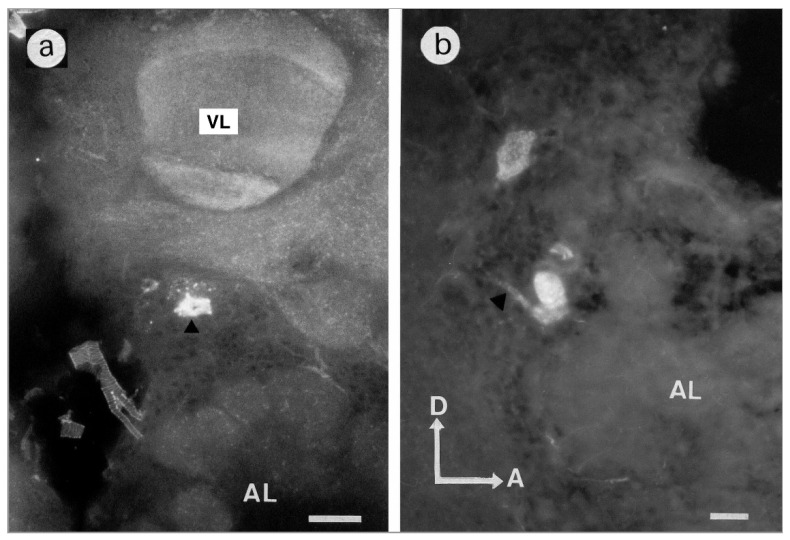
BODIPY^®^ FL Pirenzepine binding sites of the honeybee brain. The fluorescence is indicated as brightness of the micrograph. (**a**): The right side of the brain in a frontal section with the vertical lobe showing stratification. The black arrowhead indicates a stained soma of G1 cluster. Scale = 50 μm. (**b**): sagittal section through G2 cluster, arrowhead indicates the primary neurite of a cell. Scale = 25 μm. Abbreviations as in Figure 1b.

**Figure 3:**
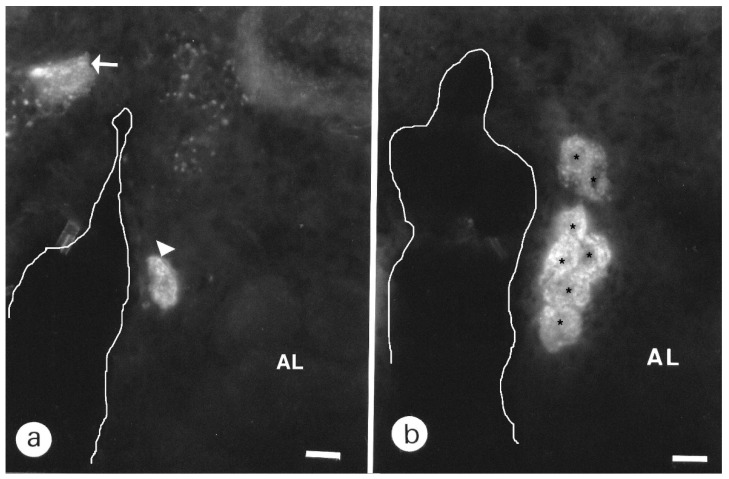
(**a**): Frontal section of the antennal lobe showing G2 somata (arrowhead) at the inner border and G1 cluster (arrow). (**b**): Asterisks indicate cell bodies of G2 cluster on a frontal section. Scale = 25 μm. The white line represents the boundary of the antennal lobe. Abbreviations as in Figure 1b.

Pirenzepine binding sites were indifferently detected in neuropilar regions and somata. The exact spatial location of six groups of somata was determined in the antero–posterior and ventro–dorsal axis (Figure 1a, Table 1). Cell bodies in the range of 20 µm in diameter were generally stained with intense fluorescent spots. An intracellular dark area was sometimes visible, corresponding to the nucleus (Figure 2 and Figure 3b).

Three groups of stained somata called G1, G2 and G3 surrounded the glomeruli of antennal lobes. Cell bodies could be identified in the median rind of the antennal lobe. The G1 group comprised four somata located in the dorsomedial region of the antennal lobe (Figure 1b and Figure 2), distributed at a depth from 138 to 206 μm (Table 1). A weak signal was still observed in the G1 somata group in the displacement experiment with unlabeled scopolamine 10^−2^ M applied over two hours before 10^−6^ M BODIPY^®^ FL pirenzepine (Figure S1c). The G2 cluster was localized in the median rind of the antennal lobe at a depth of 215 to 294 µm (Table 1) and consisted of seven somata (Figure 1b and Figure 3a,b). One stained cell sent its primary neurite outside the antennal lobe (Figure 2b). This cell was located in the area of the octopaminergic pathways previously described by [24]. Dorsally to the antennal lobe, at the depth of 52 µm, G3 likely consists of unique cells. Scarce and weak fluorescent points could be observed in glomeruli at a higher magnification (Figure 2a).

#### 3.1.2. Mushroom Bodies

**Figure 4:**
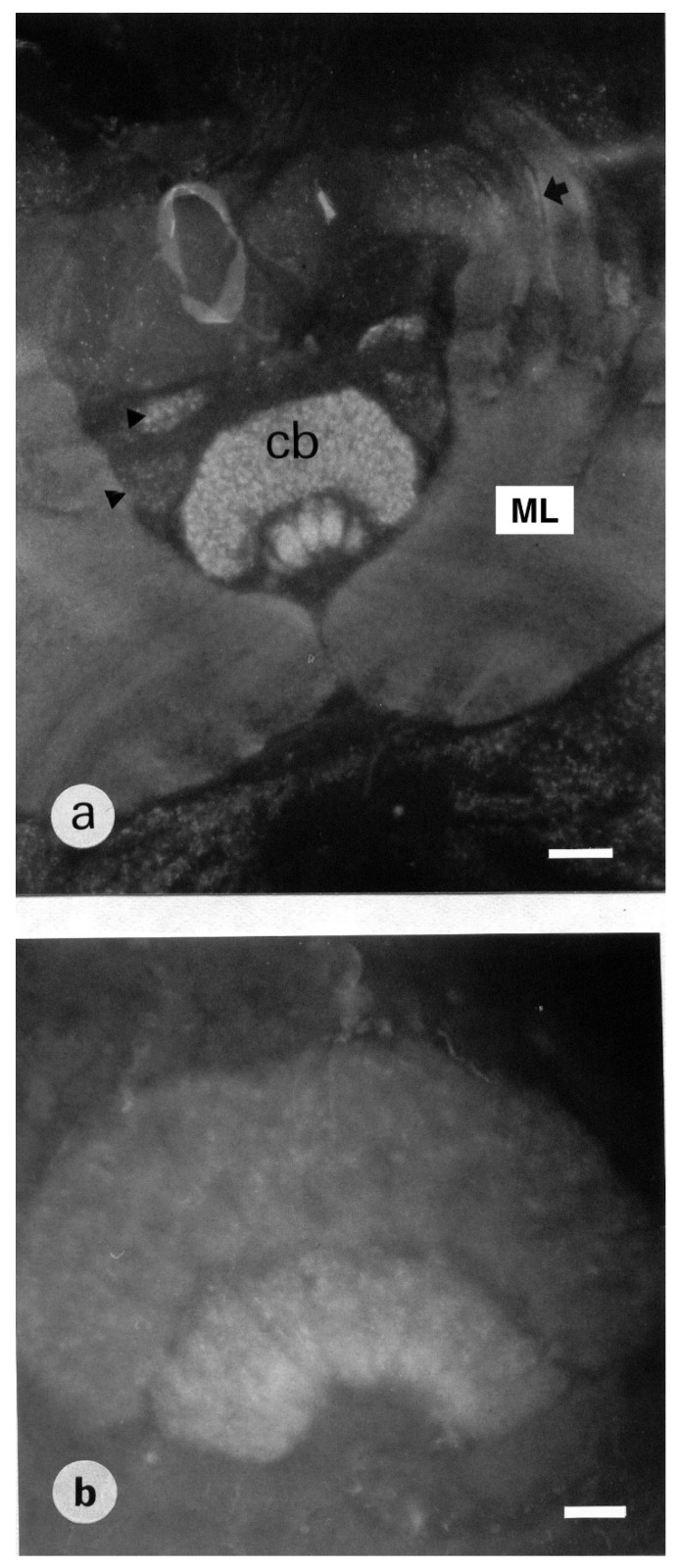
Frontal sections of the mushroom bodies peduncle (**a**) and central body (**a**,**b**). (**a**): arrowheads indicate arborizations surrounding the anterior optic tract. Note the parallel running fibers in the peduncle (arrow). Scale = 50 μm. (**b**): non-specific binding sites in the central body after preincubation with 10^−2^ M pirenzepine. Note the remaining staining in the lower division. Scale = 25 μm. Abbreviations as in Figure 1b.

Vertical lobes appeared as stratified structures. A diffuse staining was observed at the most anterior part of the vertical lobe at a depth of 50 μm. The heaviest labeling was seen in the ventral layer of the vertical lobe (Figure 1b and Figure 2a). Around the vertical lobe, PZ binding sites were also observed (Figure 2a). The medial lobes showed horizontal and vertical fluorescent stratifications, corresponding to fibers in the frontal and transversal sections (Figure 1b and Figure 4a). However, Kenyon cells somata in the lip and collar regions of the calyces were not stained (Figure S2a,b). At a depth of 500 µm, a weak labeling was observed beneath the lateral calyces in a somata zone (Figure 1b).

#### 3.1.3. Central Complex

The most fluorescent neuropil was the central body (Figure 1b and Figure 4a) with the lower layer brighter than the upper layer. BODIPY^®^ FL PZ binding sites declined when sections were coincubated with unlabeled PZ (Figure 4b). This weak labeling was still present on permeabilized slices treated with Triton-×100 (data not shown). To both sides, the central body was flanked by fluorescent fibers surrounding the anterior superior-optic tract (Figure 4a). This tract was devoid of fluorescent PZ, but fluorescence was detected in the horns of the protocerebral bridge in a more posterior level (Figure 4a and Figure S2b).

#### 3.1.4. Optic Lobes

**Figure 5:**
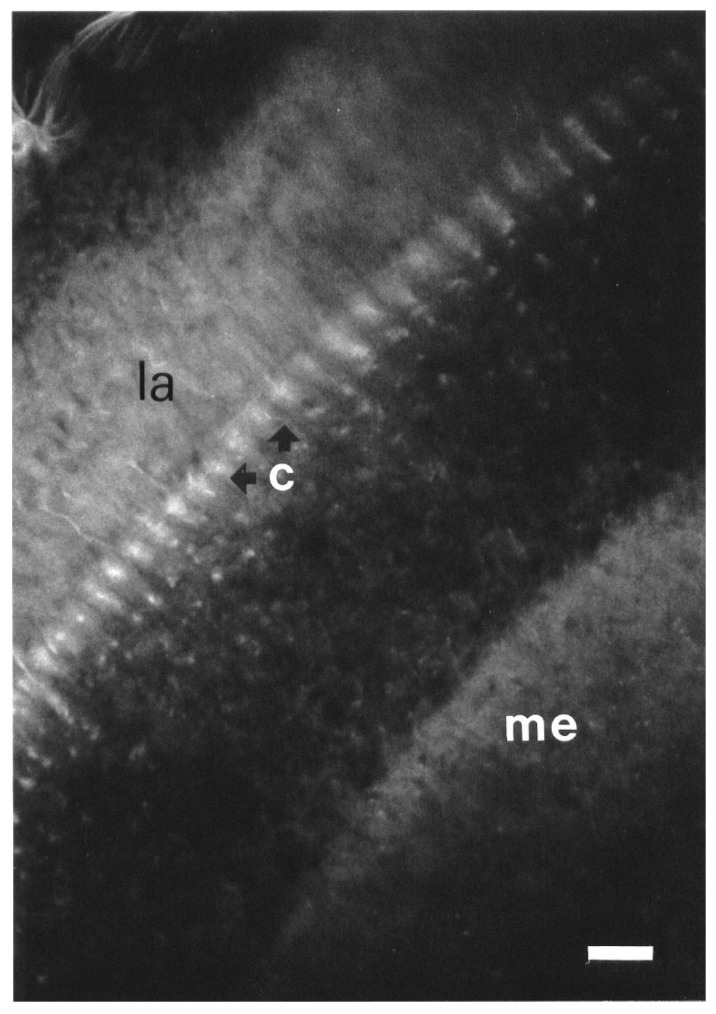
BODIPY^®^ FL Pirenzepine binding sites in the optic lobe in a frontal section. The monopolar cell bodies (arrows) of the C-layer (c) is labeled. Scale = 25 μm. Abbreviations as in Figure 1b.

The C-layer was fluorescent, and the outer border of the medulla was weakly labeled (Figure 5). In the upper part of the lamina, strongly fluorescent points were visible. The outer border of the lobula was also fluorescent.

#### 3.1.5. Subesophageal Ganglion

**Figure 6:**
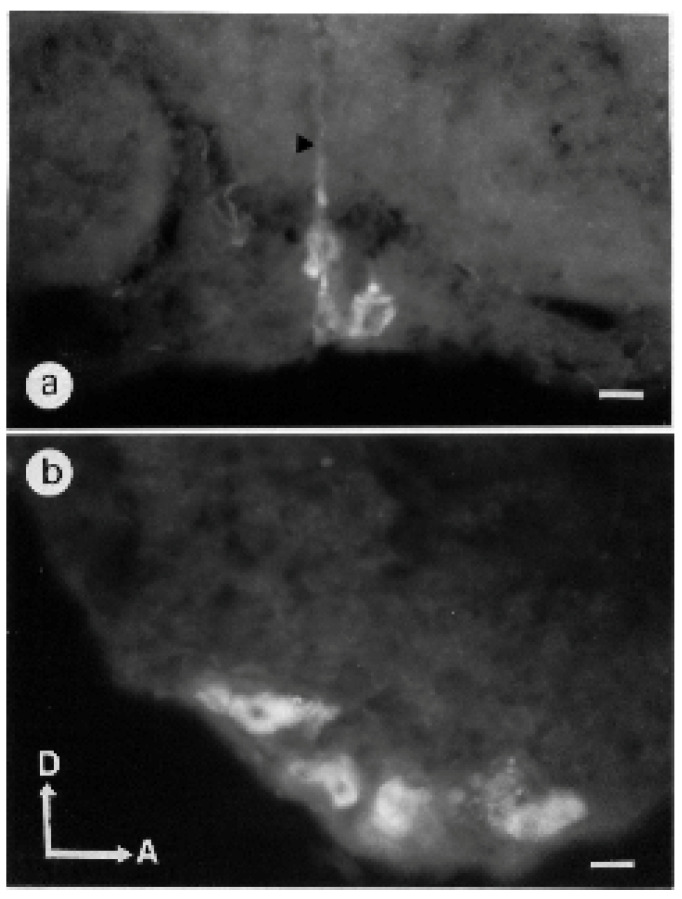
(**a**): Frontal section of the subesophageal ganglion showing ventral median somata exhibiting BODIPY^®^ FL Pirenzepine binding sites. The arrowhead indicates the primary neurite of two somata. Scale = 25 μm. (**b**): sagittal section through the midline of the subesophageal ganglion shows 4 stained cells. Scale = 25 μm.

No clear labeling was identified in the neuropilar area. Cell bodies were visible at the periphery, in the ventral medial part. One or two cell bodies were identified in the lateral part of the subesophageal ganglion corresponding to G4 (Table 1). A group of three somata dorsally sending their primary neurite was visible in the medial part (Figure 6a). The G5 cluster contained three groups of cell bodies each containing four to five somata (Figure 6b). With respect to the position of their somata, some stained neurons can be named ventral unpaired median (VUM) neurons (Figure S2c).

### 3.2. Behavioral Tests

**Figure 7:**
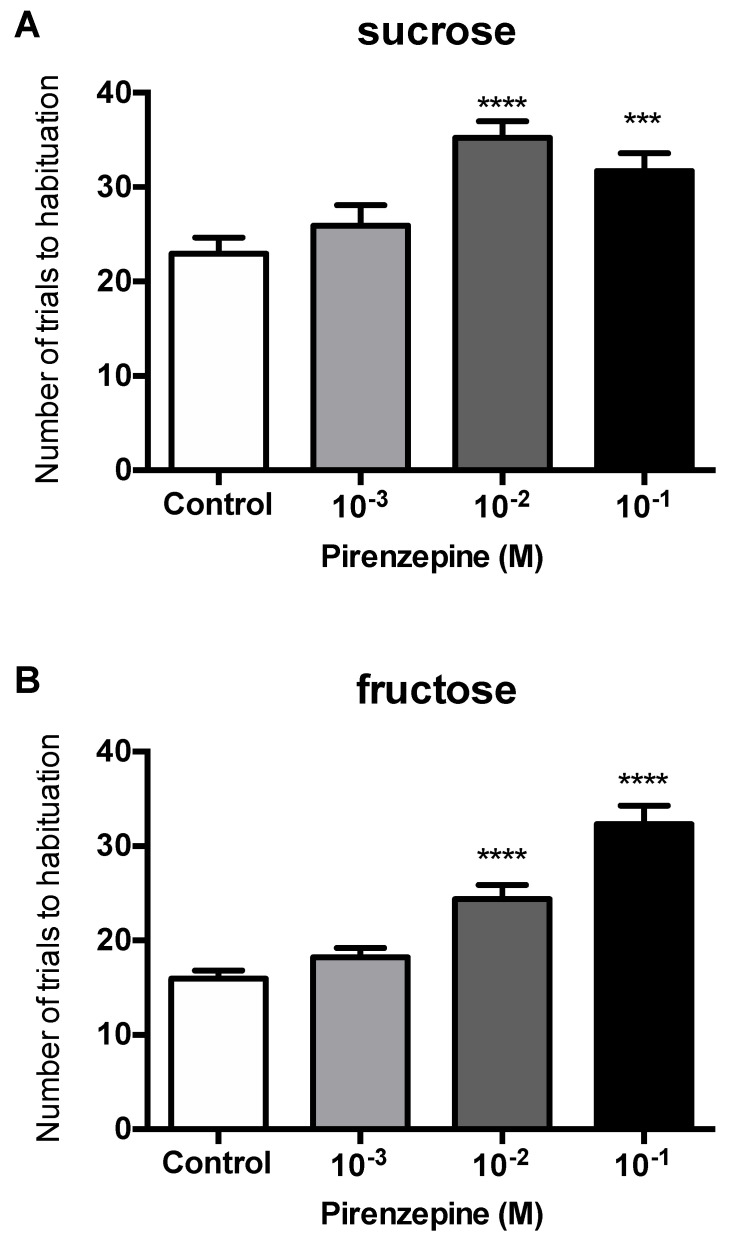
Effects of pirenzepine (PZ) on habituation after antennal stimulations by sucrose (**A**) or fructose (**B**). Mean ± SEM of the number of trials before habituation for bees tested with sucrose and treated with PBS (n = 21), PZ 10^−3^ M (n = 17), PZ 10^−2^ M (n = 20) and PZ 10^−1^ M (n = 20). Mean ± SEM of the number of trials before habituation for bees tested with fructose and treated with PBS (n = 16), PZ 10^−3^ M (n = 15), PZ 10^−2^ M (n = 16) and PZ 10^−1^ M (n = 15). *** and **** indicate a significant difference for *p* < 0.001 and *p* < 0.0001 compared to the control corresponding to each sugar.

Sugars (sucrose and fructose) led to the habituation of the PER after repeated antennal stimulation and the treatment has a significant effect whether with sucrose (F_(3, 74)_ = 9.599, *p* = 0.00002) or with fructose (F_(3,59)_ = 30.768, *p* = 4.21278 × 10^−12^) (Figure 7). Honeybees injected with PZ (10^−2^ M and 10^−1^ M) required more antennal stimulation to reach the habituation criterion compared to the controls, either with sucrose (PZ 10^−2^ M: post hoc Scheffe test = 4.84294, *p* = 0.00013 and PZ 10^−1^ M: *t*-test = 3.56918, *p* = 0.00798) or with fructose (PZ 10^−2^ M: post hoc Scheffe test = 4.70883, *p* = 0.00028 and PZ 10^−1^ M: post hoc Scheffe test = 8.54084, *p* = 2.28158 × 10^−10^). Nevertheless, analyses of the results of the habituation test with both sugars did not show any significant difference between the control group and the PZ group at 10^−3^ M (*t*-test, ns). The number of trials required for habituation was lower with fructose than with sucrose, regardless of whether the injection was of saline or PZ. Individual values for each sugar are listed in Table S1. Indeed, the number of antennal fructose stimulation to reach the criterion was 25.00 ± 1.51 and 32.38 ± 1.83 in 10^−2^ M and 10^−1^ M for the PZ groups, respectively, compared to 15.94 ± 0.86 for the control group. Meanwhile, these numbers increased when sucrose was used as an antennal stimulus and were of 34.50 ± 1.79 and 31.30 ± 1.87 stimulations for 10^−2^ and 10^−1^ M in the PZ groups versus 22.33 ± 1.58 stimulations for the control group.

**Figure 8:**
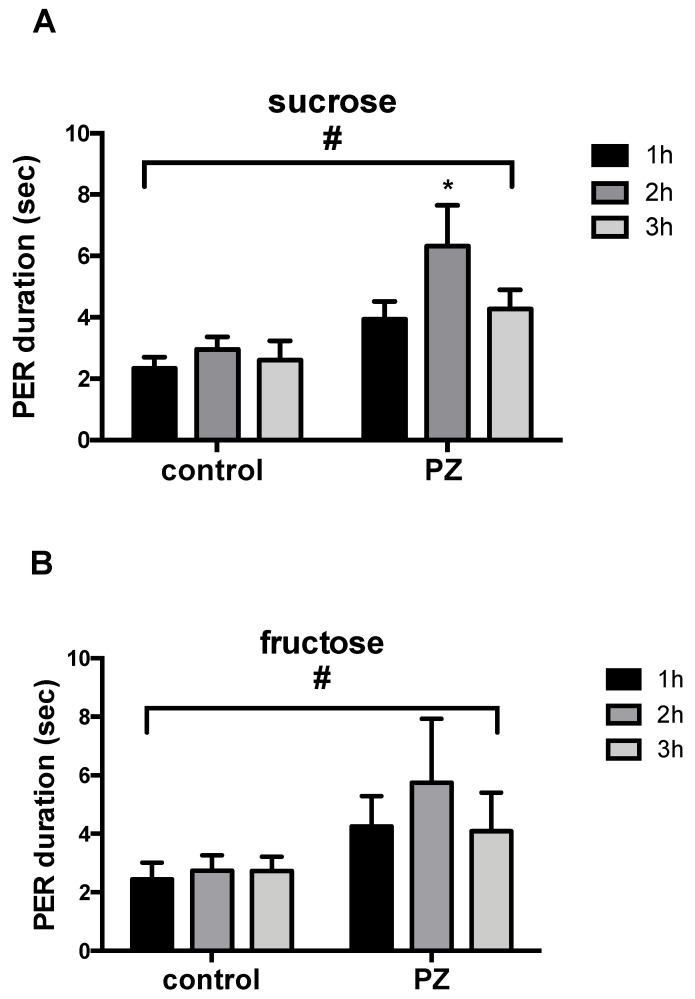
Measurement of PER duration after antennal stimulation with sucrose (**A**) or fructose (**B**) 1 h, 2 h and 3 h after injection of PBS or pirenzepine (10^−3^ M). Histograms show mean ± SEM. # *p* < 0.05, two-way ANOVA, * *p* < 0.05 Scheffe test.

The duration of the PER was significantly increased by PZ treatment (Figure 8, Table S1), regardless of which sugar was used as an antennal stimulus [sucrose (MS = 123.10260, *F* = 13.58098, *p* = 0.00037) and fructose (MS = 42.22409, *F* = 5.37831, *p* = 0.02653)]. A significant increase in PER duration elicited by sucrose was observed 2 h after PZ brain injection (Scheffe test = 3.12297, n = 19, *p =* 0.00231). Indeed, bees receiving PZ at 10^−3^ M extended their proboscis for 6 s, compared to 3 s for the control bees. However, the test with fructose did not reveal any significant increase in PER duration at 1, 2 and 3 h after injection (*t*-test, ns). Individual values and statistical analysis for each sugar are listed in Table S1.

The initial response of bees to the 50% sugar solution was not found to be significantly different between injected groups (Figure S3). The four groups stimulated by sucrose (χ^2^ = 0.8209, *p* = 0.8445) or fructose (χ^2^ = 1.982, *p* = 0.5761 showed similar responses. Pirenzepine has no effect on the PER tested before habituation.

After the honeybee attained the habituation criterion the sugar was applied to the contralateral antenna. The percentage of bees responding to the PER was more than 81% in each group (Figure S4). There were no statistically significant differences among the groups with sucrose (χ^2^ = 0.9954, *p* = 0.8024) and fructose (χ^2^ = 1.063, *p* = 0.7861) stimulations. The analysis does exclude the possibility of motor fatigue in repetitive PER after PZ treatment.

The count of the dead honeybees carried out 24 h after the injection did not reveal lethal effects of the molecule, even at the highest concentration of 10^−1^ M of PZ (Figure S5). In the control group stimulated with sucrose, the survival rate was of 80% and it was of 73% with fructose. The mortality rates of bees injected with PBS and PZ 10^−3^ M, 10^−2^ M or 10^−1^ M have not been found to be significantly different when stimulated by sucrose (χ^2^ = 0.7906, *p* = 0.8517) or fructose (χ^2^ = 7.24, *p* = 0.0644). 

## 4. Discussion

We have examined the distribution of BODIPY^®^ FL PZ binding sites in the brain of the honeybee. The data presented provide a detailed analysis of fluorescent cell bodies and neuropilar structures. Several groups of somata were identified in the antennal lobes and in the SOG, whereas fibers were stained in the peduncles of the MBs, the central complex and the optic lobes. This distribution indicates that putative mAChRs can be found on different parts of the neurons, on the cell bodies where they likely modulate the activity of the cell and along the fibers or at their end, evoking a role in synaptic transmission. Our results indicate the presence of PZ binding sites on the neurons of the honeybee brain. The affinity for a muscarinic antagonist was previously determined in honeybees with [^3^H]-QNB [19]. The mAChRs blocked by PZ could be of a different group from QNB-sensitive mAChRs. Indeed, the pharmacology of muscarinic binding sites in insects seems to be very complex [25]. In *D. melanogaster*, the A-type is blocked by QNB, while the B-type is not blocked by this antagonist [4].

The first autoradiographic distribution of insect mAChRs using the non-selective antagonist [^3^H]-QNB was performed on the cockroach brain [26]. In this study, it was striking to observe that binding sites are restricted to calyces. In addition to species differences, this mismatch supports the hypothesis that PZ binding sites are not stained by [^3^H]-QNB; PZ has a low affinity for these binding sites (Ki = 200 nM). The use of an anti-peptide antibody raised against a cloned *Drosophila* mAChR [27] shows an intense staining in antennal lobes glomeruli and somata [28,29]. A weak additional staining occurs in mushroom bodies [28]. Staining obtained with BODIPY^®^ FL PZ on cell bodies could deal with some immuno-stained cell bodies dorsal to the antennal lobe in the mid-line of the *Drosophila*’s brain [29]. However, we never observed intense staining in glomeruli. In the studies performed on *Drosophila* and *Bombyx mori*, no staining of the central complex has been described. The polyclonal antibody used is raised against a *Drosophila* cloned mAChR expressing characteristics more relevant to the A-type mAChR: this immunocytochemical localization likely concerned more of this type of binding site. By contrast, in the lepidoptera *Mythimna separata*, immunostaining indicated the presence of the MsA-type mAChR in the central body [8]. Additionally, in the developing moth, the visualization of mAChRs by BODIPY^®^ FL pirenzepine was obtained on fibers and cells bodies of antennal cell culture [30]. This expression of mAChRs was attributed to neurons and non-neural cells [30]. Therefore, a part of the BODIPY^®^ FL pirenzepine binding sites could be expressed by glial or neurosecretory cells and could be mAChRs of the B-type. The present results suggest that PZ labeling could be expressed on octopaminergic neurons [24]. Cloning and expression analysis of a putative mAChR (PmAChR) from the ant *Polyrhachis vicina* indicated that the PmAChR transcript was present in different clusters of the ant brain, including all the subpopulation of Kenyon cells [31]. We observed a scarce muscarinic-staining pattern with BODIPY^®^ FL pirenzepine in comparison to the patterns of muscarinic binding sites in MB previously reported in insects.

The existence of somal muscarinic receptors antagonized by PZ has been reported in the locust and the cockroach [11,32]. For example, the muscarinic agonist arecoline induces a slow muscarinic depolarizing current in DUM neurons, a response that is antagonized by PZ [12]. The existence of functional muscarinic receptors is questioned in the honeybee. Electrophysiological recordings of Kenyon cells of pupae demonstrated that the muscarinic antagonist atropine inhibited the ACh-induced current through the nAChRs [33].

Our experiment shows that the duration of the PER increased after PZ injection, regardless of the sugar used as an antennal stimulus. This increase could be associated with a modulation of the motor patterns controlling the mouthparts. The control of motor and rhythmic activities via an influence on mAChRs has been demonstrated in various insect ganglia preparations [9]. Upon application of the muscarinic agonist pilocarpine in the locust, an activity pattern from the mandibular opener motor nerve was recorded which was coupled with the mandibular motor pattern [34]. Additionally, S. Okada et al. [35] demonstrated that pilocarpine induces rhythmic activity of the antennal motor system in the cockroach *Periplaneta americana*. In the grasshopper, a role for mAChRs in the central complex was found to control sound production and singing behavior [36]. The injection of muscarine in the central complex resulted in the prolonged duration of stridulation activity and the highest level of arousal [37]. 

Behavioral approach in the honeybee has shown that the cholinergic pathways into the brain are widely involved in learning and memory processes. The amnesic effect of scopolamine on a one-trial olfactory conditioning [16] was confirmed with intracranial injections of atropine. However, PZ injections had no effect on the acquisition, consolidation and retrieval of the one trial olfactory conditioning task [18]. The absence of PZ binding sites on Kenyon cells and the lip part of the calyces could explain why PZ was not able to affect olfactory memory [18].

Data presented here provide support for a role of mAChRs sensitive to PZ in a non-associative form of learning. Sucrose is considered a stronger phagostimulant than fructose, and bees are more likely to learn to associate an odor with sucrose as a reward than with fructose [21]. By contrast, in the habituation test that we used, learning is easier with fructose than with sucrose. Indeed, it was observed that the number of trials required for habituation was lower with fructose than with sucrose. This observation suggests that PZ-sensitive mAChRs are expressed in neurons mediating food-induced arousal. Such a function in food arousal was also devoted to octopaminergic neurons in the honeybee brain [22]. 

## 5. Conclusions

The results obtained in our study suggest that, in the neural networks supporting habituation, mAChRs sensitive to PZ are present on some VUM neurons located in the SOG and that PZ, by modulating their activity, slow the habituation of the PER.

## Figures and Tables

**Table 1:** Estimation of the location of stained cells bodies. The depth is calculated from the surface of the antennal lobe in the antero–posterior axis on frontal sections, the height is calculated from the lower part of the subesophageal ganglion in the ventro–dorsal axis on horizontal sections as indicated on Figure 1c. G1 to G4 clusters are present on each brain hemisphere, G5 and G6 are on the midline of the subesophageal ganglion. The values represent the mean (µm) and SEM, the number of brains sectioned in one plane (n).

	Antennal Lobe			Subesophageal Ganglion		
Group	G1	G2	G3	G4	G5	G6
Number of somata	4	7–8	1	1	10–15	2
Anterior–posterior: from (μm)	138 ± 15	215 ± 14	52 ± 3	356 ± 12	432 ± 11	750
to (μm)	206 ± 17	294 ± 17	ND	ND	592 ± 22	825
n	19	18	7	9	15	2
Ventral–dorsal: from (μm)	530	364 ± 16	600	ND	56	256
to (μm)	656	432	ND	ND	160	349
n	3	6	1	9	4	6

## Data Availability

The data presented in this study are available in article and supplementary material.

## Supplementary Materials

The following supporting information can be downloaded at: https://www.mdpi.com/article/10.3390/insects13090806/s1, Figure S1: Competitive displacement experiments; Figure S2: BODIPY^®^ FL Pirenzepine binding sites of the honeybee brain; Figure S3: Pirenzepine has no effect on PER induced by sucrose and fructose before habituation; Figure S4: Restoration test; Figure S5: Mortality details; Table S1: Figure 7 and Figure 8-Statistics.
