# Supplementary material for: Pirenzepine Binding Sites in the Brain of the Honeybee Apis mellifera: Localization and Involvement in Non-Associative Learning
Source: Insects. 2022 Sep 5;13(9):806. doi: 10.3390/insects13090806 (PMC9504565; doi:10.3390/insects13090806)

S1 Supplementary results

Figures S1: Competitive displacement experiments

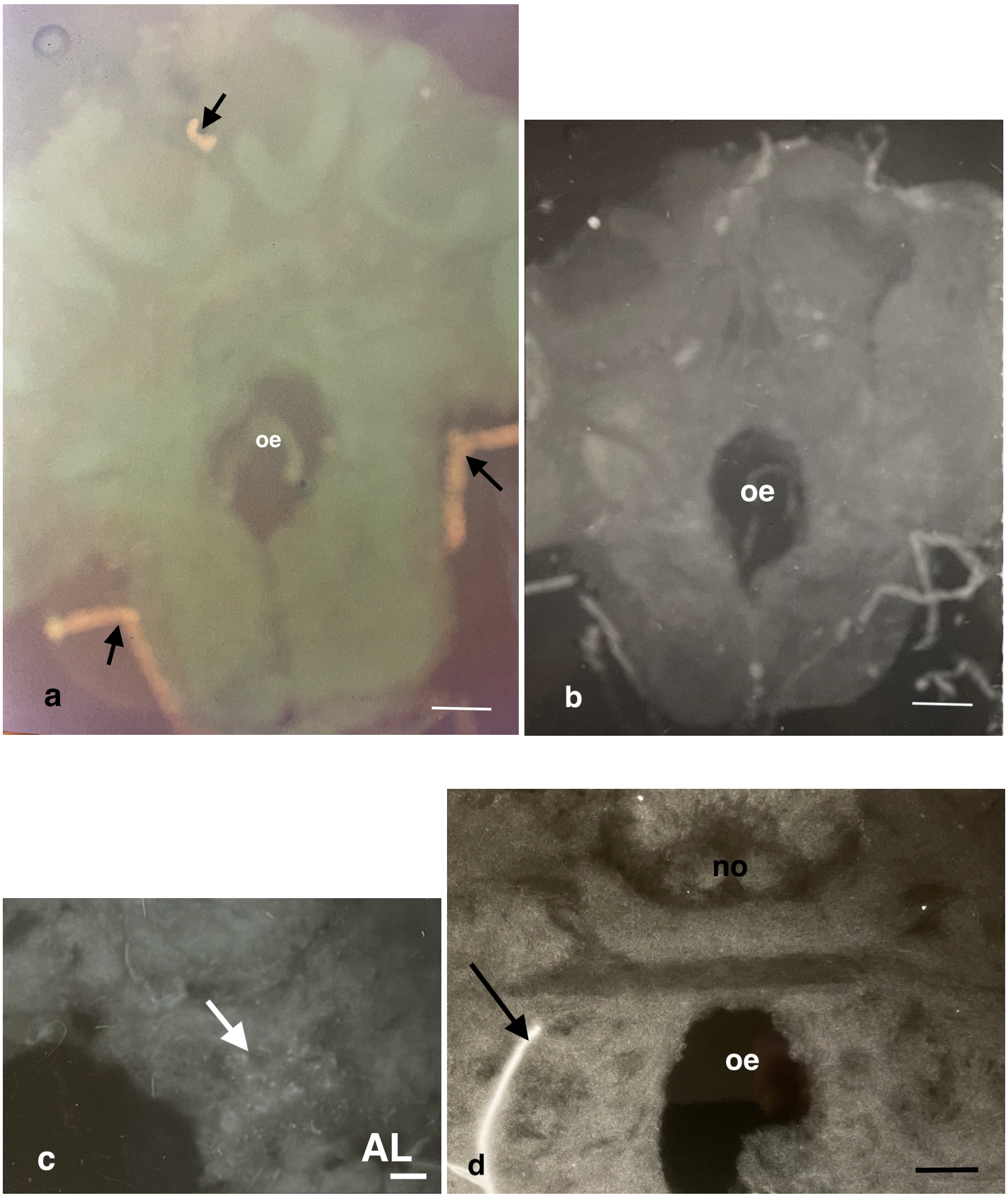

(a) Sections were initially viewed at low magnification (X4) using a FITC filter. BODIPY® FL fluorescence was green, black arrows indicate autofluorescence in yellow. (b) Digital images were captured and processed in black and white pictures for analysis. (c) A view with a X100 objective at the antennal lobe level. A weak signal was still observed in the G1 somata group (white arrow) in displacement experiment with unlabeled scopolamine 10-2 M applied during two hours before 10-6 M BODIPY® FL pirenzepine. (d): A view with a X20 objective at the noduli level of a competitive displacement experiment with unlabeled pirenzepine 10-2 M incubation before coincubation with 10-6 M BODIPY® FL pirenzepine in the presence of the unlabeled ligand. Black arrow indicates autofluorescence of trachea. AL: antennal lobe, oe: oesophagus, no: noduli.

S2 Supplementary results

Figures S2: BODIPY® FL Pirenzepine binding sites of the honeybee brain

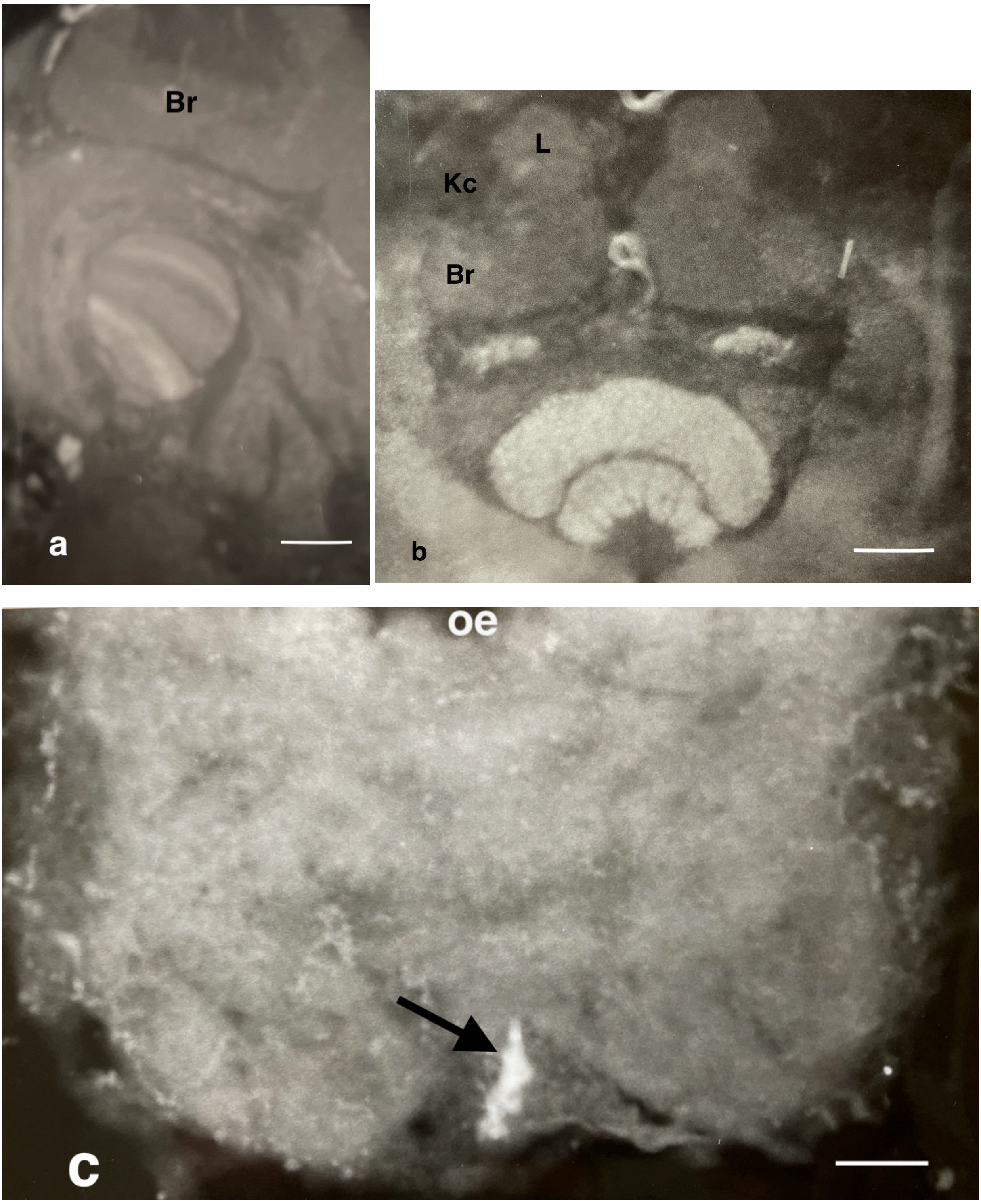

Images of the mushroom-body calyx with weak staining compared to vertical lobe (a), and central body (b). Frontal section of the suboesophageal ganglion showing VUM neuron exhibiting BODIPY® FL Pirenzepine binding sites (arrow). Br : basal ring, Kc : Kenyon cells, L : lip, oe: oesophagus. Scale=50 µm.

S3 Supplementary results

Figure S3: Pirenzepine has no effect on PER induced by sucrose (a) and fructose (b) before habituation. The PER rates of bees injected with PBS and PZ 10^{-3} M, 10^{-2} M or 10^{-1} M have not been found to be significantly different when stimulated by sucrose ($\chi^2 = 0.8203$, df=3, P=0.8445), or fructose ($\chi^2 = 1.982$, df=3, P=0.5761) before habituation.

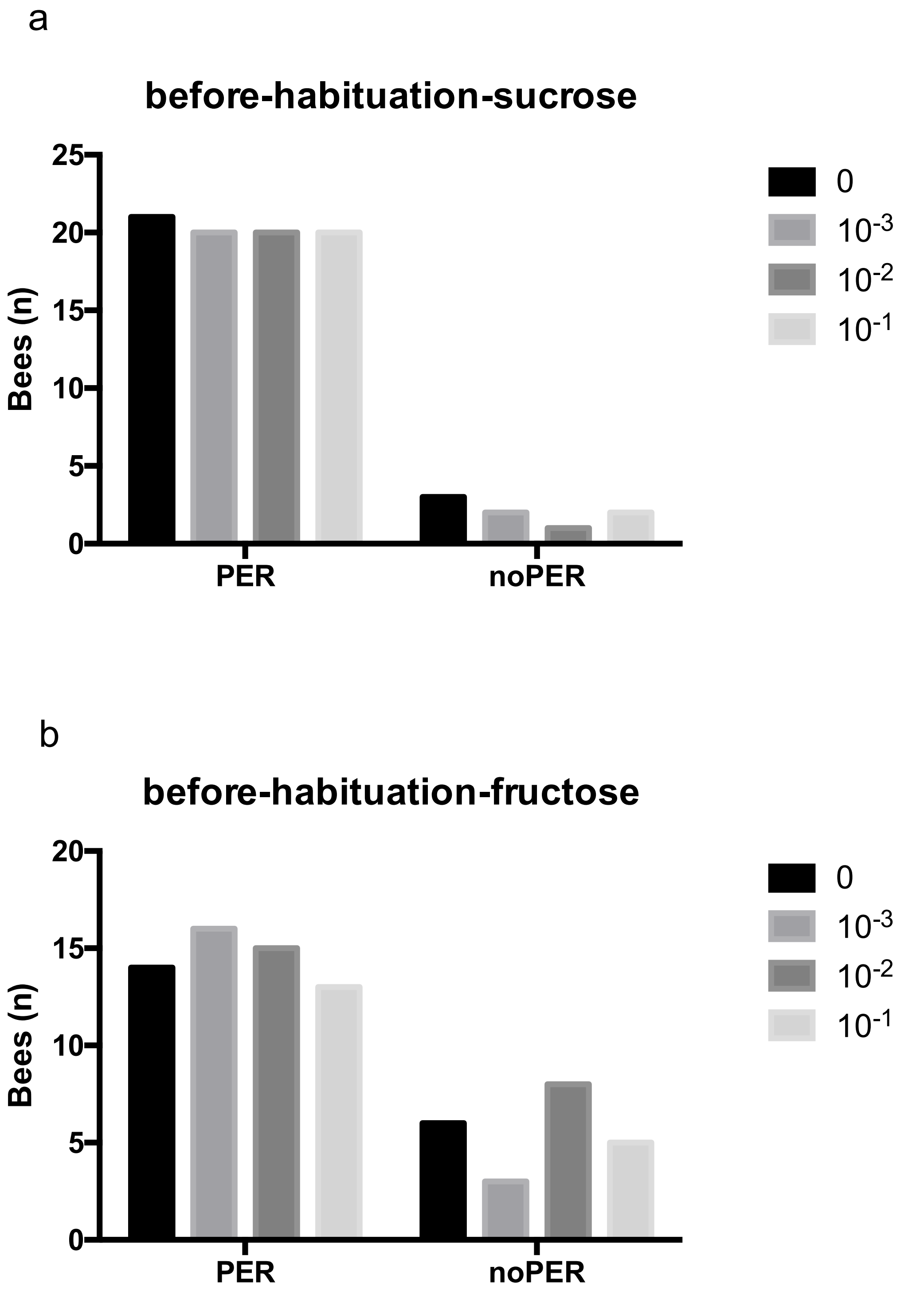

S4 Supplementary results

Figure S4 : Restoration test. After the honeybee attained the habituation criterion to exclude the possibility of motor fatigue, the sucrose (a) or fructose (b) solution was applied to the contralateral antenna. The PER rates of bees injected with PBS and PZ 10^{-3} M, 10^{-2} M or 10^{-1} M have not been found to be significantly different when stimulated contralateral antenna by sucrose (χ^2= 0.9954, df=3, P=0.8024), or fructose (χ^2 = 1.063, df=3, P=0.7861).

a

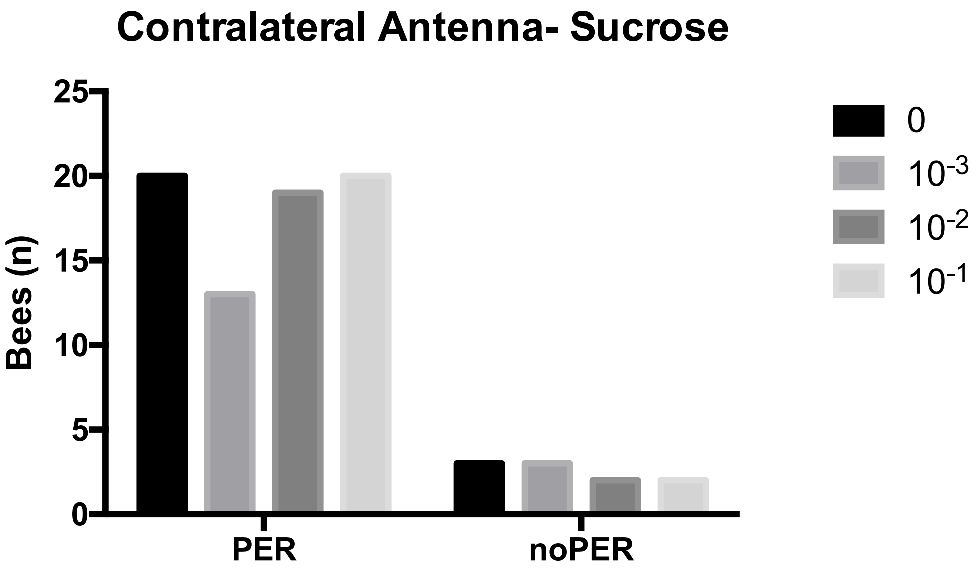

b

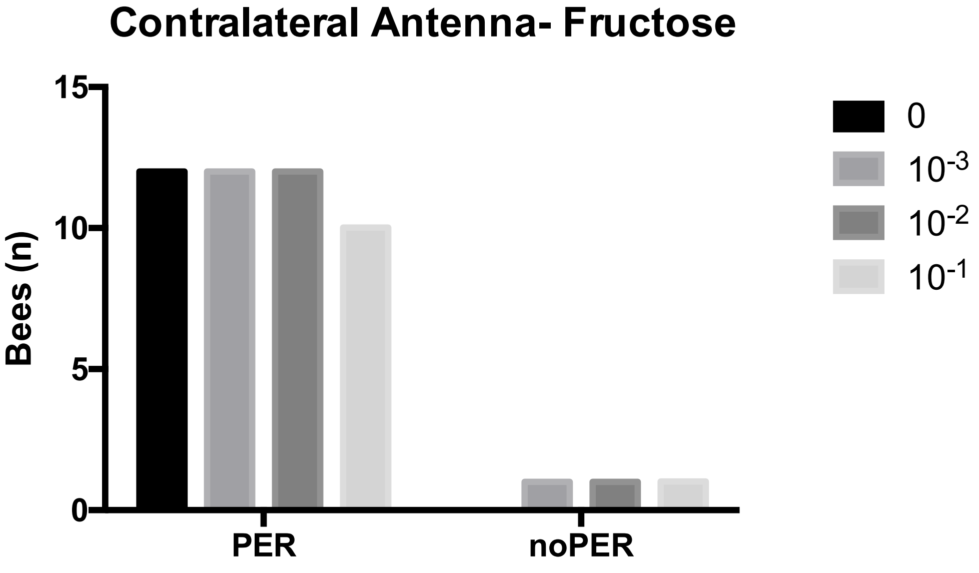

S5 Supplementary results

Figure S5: Mortality details of groups stimulated with fructose (a) or sucrose (b). The mortality rates of bees injected with PBS and PZ 10^{-3} M, 10^{-2} M or 10^{-1} M have not been found to be significantly different when stimulated by sucrose (χ^2= 0.7906, df=3, P=0.8517), or fructose (χ^2 = 7.246, df=3, P=0.0644).

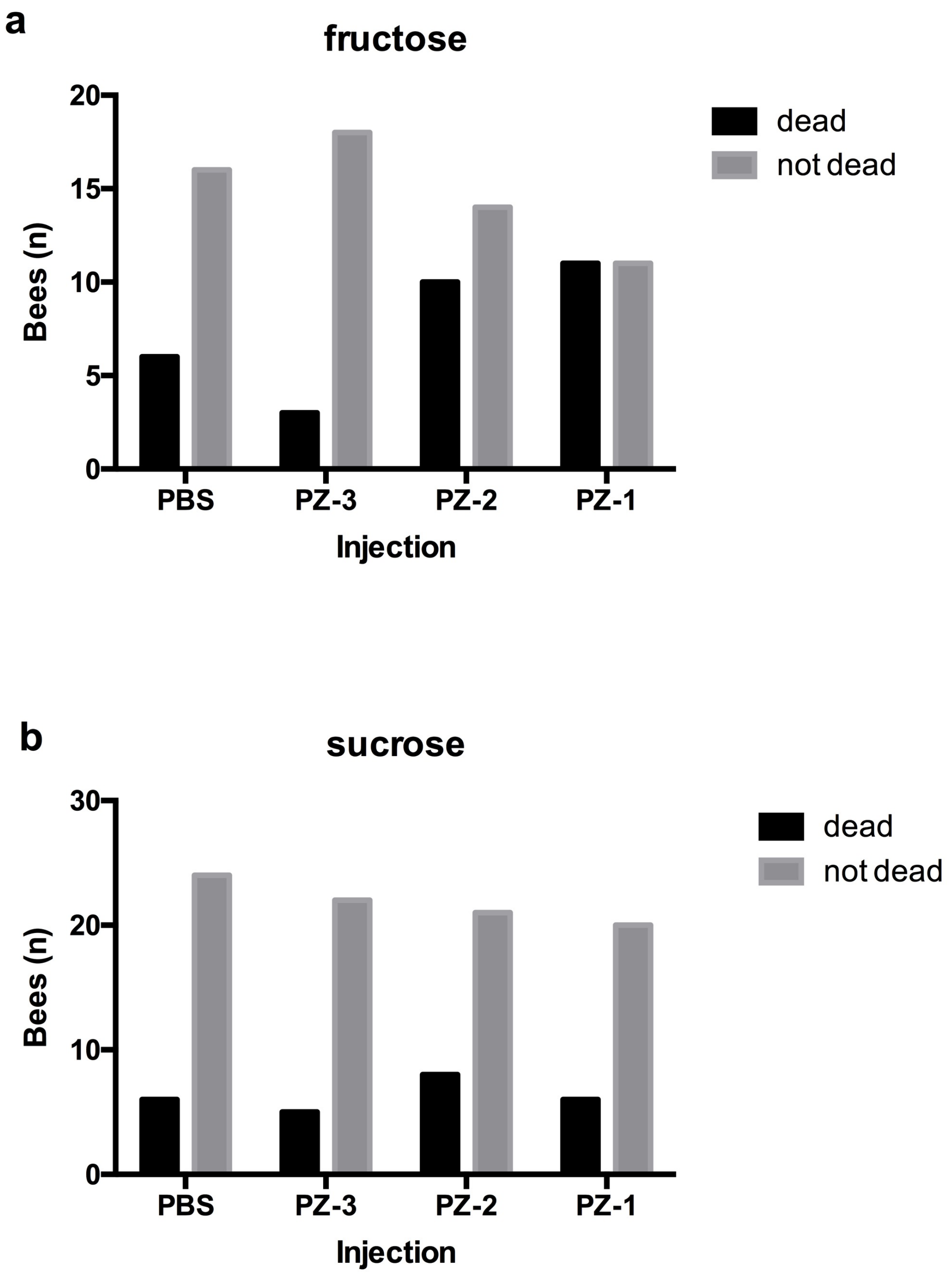

Supplement: Supplementary file 1 [file insects-13-00806-s001.zip › Supplement-Figures.pdf]
